# Dementia subtypes, cognitive decline and survival among older adults attending a memory clinic in Cape Town, South Africa: a retrospective study

**DOI:** 10.1186/s12877-023-04536-3

**Published:** 2023-12-09

**Authors:** Michael Ssonko, Anneli Hardy, Vasi Naganathan, Sebastiana Kalula, Marc Combrinck

**Affiliations:** 1 Division of Geriatric Medicine, Groote Schuur Hospital & Institute of Ageing in Africa, Cape Town, South Africa; 2 Department of Medicine, Faculty of Health Sciences, University of Cape Town, Cape Town, South Africa; 3 A Hardy Consulting, Cape Town, South Africa; 4 Concord Clinical School, Faculty of Medicine and Health, The University of Sydney, Sydney, NSW Australia; 5 Centre for Education and Research On Ageing, Department of Geriatric Medicine, Concord Repatriation General Hospital, Concord, NSW Australia

**Keywords:** Cognitive decline, Comorbidity score, Dementia subtype, Memory clinic, Mini-mental state examination, Survival, Time to event

## Abstract

**Background:**

There are no published longitudinal studies from Africa of people with dementia seen in memory clinics. The aim of this study was to determine the proportions of the different dementia subtypes, rates of cognitive decline, and predictors of survival in patients diagnosed with dementia and seen in a memory clinic.

**Methods:**

Data were collected retrospectively from clinic records of patients aged ≥ 60 seen in the memory clinic at Groote Schuur Hospital, Cape Town, South Africa over a 10-year period. Diagnostic and Statistical Manual of Mental Disorders (DSM–5) criteria were used to identify patients with Major Neurocognitive Disorders (dementia). Additional diagnostic criteria were used to determine the specific subtypes of dementia. Linear regression analysis was used to determine crude rates of cognitive decline, expressed as mini-mental state examination (MMSE) points lost per year. Changes in MMSE scores were derived using mixed effects modelling to curvilinear models of cognitive change, with time as the dependent variable. Multivariable cox survival analysis was used to determine factors at baseline that predicted mortality.

**Results:**

Of the 165 patients who met inclusion criteria, 117(70.9%) had Major Neurocognitive Disorder due to Alzheimer’s disease (AD), 24(14.6%) Vascular Neurocognitive Disorder (VND), 6(3.6%) Dementia with Lewy Bodies (DLB), 5(3%) Parkinson disease-associated dementia (PDD), 3(1.8%) fronto-temporal dementia, 4(2.4%) mixed dementia and 6(3.6%) other types of dementia. The average annual decline in MMSE points was 2.2(DLB/PDD), 2.1(AD) and 1.3(VND). Cognitive scores at baseline were significantly lower in patients with 8 compared to 13 years of education and in those with VND compared with AD. Factors associated with shorter survival included age at onset greater than 65 (HR = 1.82, 95% C.I. 1.11, 2.99, *p* = 0.017), lower baseline MMSE (HR = 1.05, 95% C.I. 1.01, 1.10, *p* = 0.029), Charlson’s comorbidity scores of 3 to 4 (HR = 1.88, 95% C.I. 1.14, 3.10, *p* = 0.014), scores of 5 or more (HR = 1.97, 95% C.I. 1.16, 3.34, *p* = 0.012) and DLB/PDD (HR = 3.07, 95% C.I. 1.50, 6.29, *p* = 0.002). Being female (HR = 0.59, 95% C.I.0.36, 0.95, *p* = 0.029) was associated with longer survival.

**Conclusions:**

Knowledge of dementia subtypes, the rate and factors affecting cognitive decline and survival outcomes will help inform decisions about patient selection for potential future therapies and for planning dementia services in resource-poor settings.

**Supplementary Information:**

The online version contains supplementary material available at 10.1186/s12877-023-04536-3.

## Background

Neurodegenerative disorders like dementia are on the rise in sub–Saharan Africa due to increased longevity leading to an increase in the numbers of older people [1]. In response, memory clinics have been established in some parts of sub-Saharan Africa to identify, investigate, and treat cognitive disorders such as dementia [2, 3]. There are few studies that have described these cohort of patients, and none that we are aware of that have reported out-patient longitudinal data. Memory clinic or hospital-based studies on people living with dementia in Africa have usually been small or have had a cross-sectional design [2–4].

Kalula et al.described a cohort of patients seen regardless of age in a memory clinic in Cape Town, South Africa. Within a period of five years, 305 people were seen of whom 74% had dementia [2]. Of these 44% had Major Neurocognitive Disorder due to Alzheimer’s disease (AD), 28% Major Vascular Neurocognitive Disorder (VND), and 15% mixed Alzheimer’s and vascular dementia. Thirteen percent had other forms of dementia, namely Dementia with Lewy bodies (DLB), Parkinson disease-associated dementia (PDD), fronto-temporal dementia (FTD), HIV-associated dementia, alcohol-related dementia, history of previous head injury and undetermined forms [2]. In this study, however, dementia diagnoses were based on clinicians’ impressions rather than standardized diagnostic criteria. In 2011 a Nigerian hospital-based study profiled dementia phenotypes of 108 patients who were inpatients over a 10-year period [4]. Of these 57.4% were diagnosed with AD, 16.7% VND, 3.7% mixed dementia, 3.7% FTD, 2.8% DLB, 2.8% alcohol related dementia, 0.9% PDD and undetermined subtypes 12% [4]. None of the memory clinic studies we reviewed that were conducted in Africa reported rates of cognitive decline or mortality data.

Mini-mental state examination (MMSE) scores have been used to determine cognitive decline in studies conducted in Western and Asian memory clinics [5–7]. A retrospective chart review of a cohort of people seen in two University Alzheimer’s Disease centres in the USA showed an average annual MMSE decline of 3.2 points in AD and 4.7 points in FTD [5]. A mainly European multi-centre study found mean annual MMSE score declines of 2.1 points with DLB, 1.6 points for AD and 1.8 points for PDD [6]. A memory clinic study in the Republic of Korea comparing AD, VND and PDD subtypes showed more rapid decline in patients with AD compared with the others [7]. Factors like age of symptom onset, level of education, and cardiovascular risk factors have also been shown to predict rates of decline [8–10]. Gerritsen et al. showed that neuropsychiatric symptoms were associated with higher rates of cognitive decline [11].

Dementia subtypes and rates of cognitive decline appear to influence survival outcomes in dementia [12, 13]. Slower rates of cognitive decline and longer survival have been shown in Alzheimer’s dementia compared with DLB and FTD [13, 14]. A Californian study, where type of dementia was confirmed by autopsy, found a survival from time of diagnosis of 4.2 years for FTD compared to 6 years for AD [13]. In this cohort, FTD had a higher cognitive decline of mean annual rate of 6.7 points compared to AD with 2.3 points [13]. A study of people seen in memory clinics in Sweden with a mean follow-up of 2.5 years found that low baseline MMSE, male gender, higher number of medications, institutionalization, and age were associated with increased mortality after dementia diagnosis [15]. A retrospective study carried out in three Italian dementia out-patient clinics found age, gender and functional status to be the main determinants of patient survival [16]. An Australian study with participants from nine memory clinics found that 57.4% of 779 patients with dementia had died within eight years [17]. In this study, greater deterioration in dementia severity and functional impairment over time predicted mortality independent of baseline levels [17]. A study in specialised outpatients’ dementia clinics in Spain found AD to have the best survival while subtypes like Parkinson-Plus Syndromes and dementia due to multiple aetiologies sub-types had the worst prognosis [18]. A Dutch study carried out among patients with young onset dementia in specialised centres found AD to have a worse survival compared with VND subtype [19]. The same study found a trend of decreased survival for the participants with AD compared with FTD [19].

There are, to our knowledge, no published longitudinal studies of patients with dementia from memory clinics in sub-Saharan Africa that have characterized the subtypes, cognitive decline, survival outcomes and predictors of survival. Dementia subtypes have distinctive natural histories. A precise diagnosis may lead to a better understanding of prognosis. Data regarding rates of cognitive decline and survival of the different dementia subtypes have also largely been derived from populations in the developed world. Accurate clinical diagnosis is especially important in resource poor settings where expensive investigations are not readily available. With the future advent of potential specific drug therapies, an accurate diagnosis as well as a knowledge of probable survival outcomes of dementia subtypes may be useful. A knowledge of the characteristics of patients seen in memory clinics and their longitudinal trajectories can also be used to further develop these clinics and services of older people with dementia. The aim of this study, using data collected on older adults who attended the memory clinic at Groote Schuur Hospital in Cape Town, was to determine the proportions of the different dementia subtypes, the rates of cognitive decline, trajectories of decline of the different dementia sub-types, and to determine whether their correlations exist between dementia subtypes and survival rates.

## Methods

### Study design and procedure

Data were obtained from patients’ memory clinic case records using a standardized data collection form for patients aged 60 and above seen during a 10-year study period from 1^st^ January 2010 to 31^st^ December 2019.

The memory clinic is a sub-specialist outpatient clinic of Groote Schuur Hospital in Cape Town, South Africa. The clinic is held weekly, and its clinical staff consists of a team of geriatric medicine physicians, neuropsychiatrists, a neurologist, neuropsychologists, and sub-specialty trainees. Patients are referred from general practitioners, family physicians, medical officers from community health clinics and state or private specialists in the Western Cape region. The catchment population is mainly urban with a mix of socioeconomic status. The clinical staff triage the referrals into those who would be seen in the memory or geriatric clinics. In general, patients with higher MMSE scores (≥ 15) are more likely to be testable using the full neuropsychology battery and are therefore seen in the memory clinic. Those with lower MMSE scores are seen in a geriatric clinic, where a less detailed and more focused cognitive assessment is performed. Patients are usually accompanied by a family member/caregiver who assists with collateral history. Patients undergo a full medical examination. Baseline cognitive assessments were generally administered in English by the neuropsychology team. If a patient didn’t speak English, the tests were informally translated by a tester who could speak the patient’s first language. The tests include assessing general cognitive functioning using MMSE and Montreal Cognitive Assessment (MoCA) scores. Specific cognitive abilities are assessed as follows: Learning and memory by Repeatable Battery for the Assessment of Neuropsychological Status (RBANS) list learning with delayed recall, RBANS Story Memory with delayed recall and RBANS Figure Recall. Language is assessed by verbal fluency that includes both semantic and phonemic assessments, Boston Naming test (short form), and an assessment for speech quality (i.e., clarity, difficulty in making oneself understood, and difficulty in understanding). Attention and/or working memory is assessed by digit span forward and backwards and months of the year backwards. Frontal lobe or executive functioning by trails A, MoCA Trail, trails B, CLOX 1 and 2, Luria recursive figures and hand sequence. Visuo-perceptual or spatial ability is assessed by the RBANS figure copy, and CLOX test. The RBANS battery was not validated in this memory clinic population.

Laboratory investigations to exclude reversible causes of cognitive impairment are conducted. These include tests of renal, liver, and thyroid function, serum calcium levels, serum levels of vitamin B-12, HIV, and syphilis serology tests. Patients undergo neuroimaging—usually computed tomography (CT) of the brain—but other neuroimaging modalities such as Magnetic Resonance Imaging (MRI), Single Photon Emission Computed Tomography (SPECT), and 18-fluoro deoxy glucose Positron Emission Tomography (18-FDG-PET) are occasionally done if indicated.

The multi-disciplinary team meets at the end of each clinic to discuss the patients’ likely diagnoses and plan of action. Cognitive assessments using MMSE and/or MoCA are usually carried out by the attending clinician at six-monthly intervals corresponding with the patient’s follow-up clinic visits. Once a treatment plan is agreed upon, patients are discharged back to the care of the referring centre.

For this study, a consensus diagnosis of dementia and dementia sub-type was determined by a retrospective review of hospital records by the Neurologist (MC) and the trainee sub-specialist in geriatric medicine (MS). The Diagnostic and Statistical Manual of Mental Disorders (DSM–5) criteria for Major Neurocognitive Disorder were used to determine if the patients had dementia. To determine dementia subtypes the following standard diagnostic criteria were used: National Institute of Neurological and Communicative Disorders and Stroke and the Alzheimer's Disease and Related Disorders Association for AD [20], National Institute of Neurological Disorders and Stroke and the Association Internationale pour la Recherche et l’Enseignement en Neurosciences (for cerebral vascular disease description) for VND [21], Work Group on fronto-temporal dementia and Pick’s disease for FTD [22], and consensus guidelines for the clinical and pathologic diagnosis of dementia with Lewy bodies [23]. We also used a validated set of diagnostic criteria for PDD [24, 25].

A participant was classified as having the subtype of dementia if he/she met the probable or the possible criteria as per the outlined standard diagnostic criteria above. It was possible for participants to meet criteria for two different subtypes of dementia. If a participant met two different probable or two different possible dementia diagnoses according to criteria, they were classified as having mixed dementia. If participants met the criteria for probable dementia of one type and possible dementia of another type, they were categorized as only having the probable dementia subtype (e.g., if they met criteria for probable AD and possible VND, they were categorized as AD). Table 1 shows details of how this was done using AD and VND as examples.

**Table 1:** Approach to determining a participant's dementia subtype (using AD and VND as an example)

First Dementia Subtype criteria	Second dementia subtype criteria	Dementia subtype Category for Study
Probable AD	Probable VND	Mixed Dementia
Possible AD	Possible VND	Mixed Dementia
Probable AD	Possible VND	AD
Possible AD	Probable VND	VND

The baseline visit was identified as the date the patient was first seen during the study period and met the study criteria. The education level was the highest level attained at baseline. Duration of symptoms prior to diagnosis was estimated from the earliest symptom of dementia as obtained from the patient and/or caregiver. Comorbidity scores were derived from the Charlson’s Weighted Index of Comorbidity [26]. Baseline laboratory investigations were carried out within three months before or after dementia diagnosis. Blood pressure measurements and MMSEs were carried out at baseline and subsequent clinic visits, usually with intervals of six months. Cholinesterase inhibitors were prescribed for patients who could afford to purchase them from private pharmacies since they were not available in state services. We checked for dates of death at the state registry managed by the South African Medical Research Council (MRC) for all enrolled participants on 30^th^ November 2021.

Participants with advanced dementia (unable to complete at least 50% of neuropsychology battery tests and dependent, in the absence of physical disability, on three or more basic activities of daily living), were excluded from the study.

Ethics approval was obtained from the Human Research Ethics Committee of the Faculty of Health Sciences, University of Cape Town (HREC-REF: 403/2021). Permission to conduct the study was obtained from the medical superintendent of Groote Schuur Hospital.

### Statistical analysis

#### Characteristics

Analyses were performed using Stata version 17.0 [27]. Proportions of participants diagnosed with different sub-types of dementia were expressed as percentages of the total number of participants in the study. Frequency tables and cross tabulations of sociodemographic and clinical variables were undertaken. Normally distributed data were expressed as means and standard deviations and compared using one way analysis of variance (one-way anova). Tukey or Scheffe tests were used for post-hoc comparisons to assess which group pairs differed significantly. Non-normally distributed data were expressed as median and interquartile ranges and compared using Kruskal–Wallis tests. Comparisons of categorical variables were performed using chi-square tests of independence.

#### Cognitive decline

Periods of 12-month intervals of mean MMSE scores, for participants who had more than one annual score, were used for the analysis of cognitive decline. Linear regression was used to determine the crude rates of cognitive decline, expressed as MMSE points lost per year. We used MMSE scores with mixed effects modelling to curvilinear models of cognitive change with time as the dependent variable. Different models using time to event (TTE) i.e., from onset of symptoms to death or end of study period, age at onset of dementia symptoms and age at diagnosis of dementia, were used to determine whether independent variables like gender, years of education, comorbidity score and dementia subtypes predicted cognitive decline. There was no serious deviation from assumptions of normality and constant variance.

#### Survival

Survival time was defined as time from symptom onset of dementia until the date of death. The Kaplan–Meier method was used to estimate the mean and median survival times among the different dementia sub-types. A comparison of survival rates was done by the log rank test. We used Kruskal–Wallis test to assess survival outcomes between the dementia subtype groups because of the non-normally distributed data. Post hoc comparison tests were used to assess which group pairs differed, and significant results were based on a Bonferroni adjusted level alpha of 0.005. Cox models were used to determine factors associated with survival. Potential risk factors for reduced survival and those with p-values of < 0.2 in univariate analysis, were entered into multivariate modelling. Variables were also included in the multivariate model of Cox regression with the Breslow method for ties to determine which factors at baseline predicted reduced survival. The model was found to have reasonable predictive power (using Harrell’s C concordance statistic) and there were no serious violations of the assumptions of proportionality (based on Schoenfeld residuals). The significance level was set at *p* < 0.05.

## Results

**Fig. 1:**
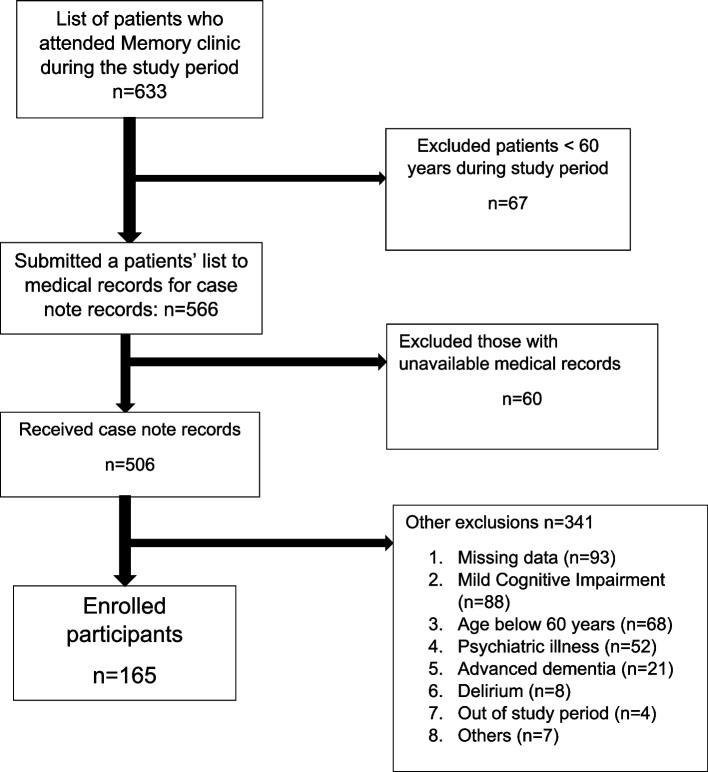
Flow chart of study participants’ cohort with dementia

A total of 633 patients attended the memory clinic over the 10-year study period. Of those, 67 who were aged less than 60 years, were excluded. A list of 566 patients was submitted to the medical records department for retrieval of their case records. Of these we received 506 (89.4%). A total of 341 patients were excluded as they did not meet the study criteria, resulting in a final study cohort of 165 participants as shown in Fig. 1. The main reasons for exclusion after reviewing the case notes on the 506 included missing data (*n* = 93), a diagnosis of mild cognitive impairment (*n* = 88), age below 60 years (*n* = 68), psychiatric illness (*n* = 52), advanced dementia (*n* = 21), delirium (*n* = 8), out of study period (*n* = 4), and other reasons (*n* = 3 seen in the Geriatric clinic, *n* = 1 no cognitive impairment, *n* = 1 metastases to the brain, *n* = 1 tuberculous meningitis and n = 1 intellectual disability).

### Dementia Subtypes

Table 2 shows the categorization of the study cohort by dementia subtypes. We classified 117 (70.9%) as having AD, 24 (14.6%) with VND, 6 (3.6%) with DLB, 5 (3%) with PDD, 3 (1.8%) with FTD, 4 (2.4%) with mixed dementia and 6 (3.6%) with other types of dementia.

**Table 2:** Frequencies of the types of dementia

Dementia sub-type	n (%)
AD	117 (70.9)
VND	24 (14.6)
DLB	6 (3.6)
PDD	5 (3.0)
FTD	3 (1.8)
Mixed Dementia	**4 (2.4)**
AD + VND	3 (1.8)
AD + DLB	1 (0.6)
Others	**6 (3.6)**
Alcohol Related	2 (1.2)
Huntington's	1 (0.6)
Brain Tumour	1 (0.6)
HSV Encephalitis	1 (0.6)
Craniopharyngioma	1 (0.6)

*Abbreviations*: *n* number, *AD* Major Neurocognitive Disorder due to Alzheimer’s disease, *VND* Major Vascular Neurocognitive Disorder*;* *DLB* Dementia with Lewy bodies*;* *PDD* Parkinson’s disease dementia, *FTD* Fronto-temporal dementia, *HSV* Herpes Simplex Virus

### Demographic and clinical characteristics

Table 3 shows the socio-demographic and clinical characteristics of the study cohort. There were more females 101 (61.2%) than males. The majority 104 (63.4%) had duration of symptoms for two or more years prior to dementia diagnosis. Most participants had a comorbidity score of 1 to 2 (40%) followed by a score of 3 to 4 (33.3%). At baseline, 39 (23.6%) had MMSE scores of 25 to 30, 89 (53.9%) MMSE scores 19 to 24 and 35 (21.2%) MMSE scores of 10 to 18. Two individuals with low MMSEs of < 10 had a diagnosis of Primary Progressive Aphasia and another with Alcohol Related Dementia. Both had fair functionality though with communication challenges and able to complete 50% of the applied test batteries.

**Table 3:** Socio-demographic and clinical characteristics of participants

*Characteristics*	*n (%)*
**Age at Diagnosis**	
60—65	30 (18.2)
66—70	39 (23.6)
71—75	40 (24.2)
76—80	35 (21.2)
81—85	16 (9.7)
> 85	5 (3.0)
**Gender**	
Male	64 (38.8)
Female	101 (61.2)
**Marital Status**	
Married	91 (55.2)
Divorced	20 (12.1)
Widowed	47 (28.5)
Single	7 (4.2)
**Years of education**	
0 to 7	39 (23.8)
8 to 12	105 (64.0)
> 12	20 (12.2)
**Duration of symptoms (months)**	
< 6	3 (1.8)
6 to 11	11 (6.7)
12 to 23	46 (28.0)
> 23	104 (63.4)
**Charlson’s Weighted Comorbidity score**	
1 to 2	66 (40.0)
3 to 4	55 (33.3)
≥ 5	44 (26.7)
**Baseline MMSE**	
25—30	39 (23.6)
19—24	89 (53.9)
10 to 18	35 (21.2)
< 10	2 (1.2)
**Vitamin B12 (pmol/L)**	
> 150	128 (77.6)
≤ 150	14 (8.5)
Not Done	23 (13.9)
**Syphilis**	
Non—Reactive	133 (80.6)
Not Done	32 (19.4)
**TSH (mlU/L)**	
0.38 to 5.33	132 (80.0)
< 0.38	6 (3.6)
> 5.33	12 (7.3)
Not Done	15 (9.1)
**HIV**	
Negative	68 (41.2)
Not Done	97 (58.8)

*Abbreviations*: *n* number, *MMSE* Mini-Mental State Examination, *TSH* Thyroid Stimulating Hormone, *HIV* Human Immunodeficiency Virus, *TSH* Thyroid Stimulating Hormone – Normal range = 0.38 to 5.33 mIU/L; Vitamin B12 – Normal range = 145 to 569 pmol/L (<150pmol/L is WHO cut off for vitamin B12 defficiency);WHO, World Health Organisation; Footnote table 3: the number with missing results are as follows: years of education (*n*=1), duration of symptoms (*n*=1)

Table 4 shows the demographic and clinical characteristics of the study cohort by dementia subtype. The mean age at diagnosis of dementia was 72.4 years and was highest for the AD group (73.6 years, SD = 7.2). There was a statistically significant difference in age at diagnosis for the dementia subtypes F (4, 154) = 3.88, *p* = 0.005. Using the Scheffe test, the mean age at diagnosis was only significantly different between AD and VND (mean difference = 5.09, *p* = 0.029). Participants with DLB or PDD had the highest age at onset of 70.9 years, SD = 6.2, F (4, 153) = 3.17, *p* = 0.016. The Tukey test indicated that the mean age at onset was only different between AD and VND (mean difference = 4.32, *p* = 0.046). The overall time since symptom onset and diagnosis was 30.6 months (SD = 23.1) and highest for FTD with 60 months, SD = 24, F (4, 153) = 2.43, *p* = 0.05. The Tukey test showed that participants with FTD tended to differ from VND (mean difference = 37.13, *p* = 0.066). The overall mean follow-up time since symptom onset was 7.2 years (SD = 3.3) and least for DLB/PDD group with 4.96 years, SD = 2.5. There was a statistically significant difference in mean follow-up time for the dementia subtypes χ^2^(4) = 15.39, *p* = 0.04.

**Table 4:** Demographic and clinical characteristics of participants according to dementia subtype

Characteristics	All (*n* = 165)	AD (*n* = 117)	VND (*n* = 24)	DLB or PDD (*n* = 11)	FTD (*n* = 3)	Mixed (*n* = 4)	F-test	*p*-value
**Age at Diagnosis (years)**								
Mean (SD)	72.4 (7.0)	73.6 (7.2)	68.5 (5.5)	73.1 (5.7)	69 (7.2)	66.3 (5.1)	3.88	0.005
^**c**^**Age at onset (years)**	**(*n* = 164)**	**(*n* = 116)**						
Mean (SD)	69.7 (7.1)	70.74 (7.2)	66.4 (5.4)	70.9 (6.2)	64 (8.7)	64.5 (4.8)	3.17	0.016
^**c**^**Time since symptom onset and diagnosis (months)**	**(*n* = 164)**	**(*n* = 116)**						
Mean (SD)	30.6 (23.1)	32.3 (22.4)	22.9 (27.4)	26.2 (16.8)	60 (24)	19.5 (13.3)	2.43	0.05
**Mean Follow-up time: Years (SD)**	7.2 (3.3)	7.8 (3.4)	5.8 (2.6)	4.96 (2.5)	7.7 (1.9)	6.3 (4.4)	15.39^b^	0.004
**Gender: n (%)**								
Male	64 (38.8)	34 (29.1)	19 (79.2)	6 (54.6)	2 (66.7)	1 (25.0)	23.55^a^	*p* < 0.001
Female	101 (61.2)	83 (70.9)	5 (20.8)	5 (45.5)	1 (33.3)	3 (75.0)	0.39^a^	*p* < 0.001
**Marital Status: n (%)**								
Married	91 (55.2)	59 (50.4)	18 (75.0)	2 (100.0)	2 (66.7)	3 (75.0)	18.45^a^	0.103
Divorced	20 (12.1)	12 (10.3)	4 (16.7)	0	1 (33.3)	1 (25.0)	0.20^a^	0.051
Single	7 (4.2)	5 (4.3)	0	0	0	0		
Widowed	47 (28.5)	41 (35.0)	2 (8.3)	0	0	0		
**Years of Education: n (%)**								
0 to 7	39 (23.8)	33 (28.5)	3 (12.5)	1 (9.1)	1 (33.3)	1 (25.0)	14.24^a^	0.076
8 to 12	105 (64.0)	69 (59.5)	18 (75.0)	9 (81.8)	0	3 (75.0)	0.21^a^	0.108
> 12	20 (12.2)	14 (12.1)	3 (12.5)	1 (9.1)	2 (66.7)	0		
**Baseline MMSE**								
Median (IQR)	21 (5)	21 (5)	23 (8)	23 (11)	19 (23)	19.5 (10.5)	3.74^b^	0.442
**Charlson's Weighted Comorbidity score**								
1 to 2	66 (40.0)	49 (41.9)	7 (29.2)	4 (36.4)	1 (33.3)	1 (25.0)	12.33^a^	0.137
3 to 4	55 (33.3)	43 (37.5)	5 (20.8)	3 (27.3)	1 (33.3)	3 (75.0)	0.20^a^	0.121
≥ 5	44 (26.7)	25 (21.4)	12 (50.0)	4 (36.4)	1 (33.3)	0		
**MAP**	**(*n* = 93)**	**(*n* = 66)**	**(*n* = 15)**	**(*n* = 6)**	**(*n* = 1)**	**(*n* = 2)**		
Median (IQR)	101.7 (16.7)	100 (16.7)	104.7 (15)	107.5 (17.3)		116 (21.3)	2.28^b^	0.684
**Vitamin B12 (pmol/L)**	**(*n* = 138)**	**(*n* = 104)**	**(*n* = 20)**	**(*n* = 7)**	**(*n* = 3)**	**(*n* = 4)**		
Median (IQR)	293 (174)	295.5 (179)	309.5 (226)	252 (67)	309 (141)	211.5 (248.5)	0.89^b^	0.927
**TSH mlU/L**	**(*n* = 150)**	**(*n* = 107)**	**(*n* = 21)**	**(*n* = 10)**	**(*n* = 3)**	**(*n* = 4)**		
Median (IQR)	1.52 (1.4)	1.44 (1.4)	1.9 (1.9)	1.1 (0.8)	1.5 (0.9)	1.6 (0.8)	4.98^b^	0.29

^a^chi-square value for the chi-square test of independence to compare two categorical variables

^b^Kruskal-Wallis tests: non-normally distributed data

^c^Missing data for duration of symptoms prior to diagnosis in a participant with AD

*Abbreviations*: *n* number, *SD* Standard Deviation, *IQR* Inter Quartile Range, *MMSE* Mini-Mental State Examination, *MAP* Mean Arterial Pressure, *TSH* Thyroid Stimulating Hormone – Normal range = 0.38 to 5.33 mIU/L; Vitamin B12 – Normal range = 145 to 569 pmol/L; AD, Major Neurocognitive Disorder due to Alzheimer’s disease; *VND* Major Vascular Neurocognitive Disorder, *DLB* Dementia with Lewy bodies, *PDD* Parkinson’s disease dementia, *FTD* Fronto-temporal dementia

### Cognitive decline

**Fig. 2:**
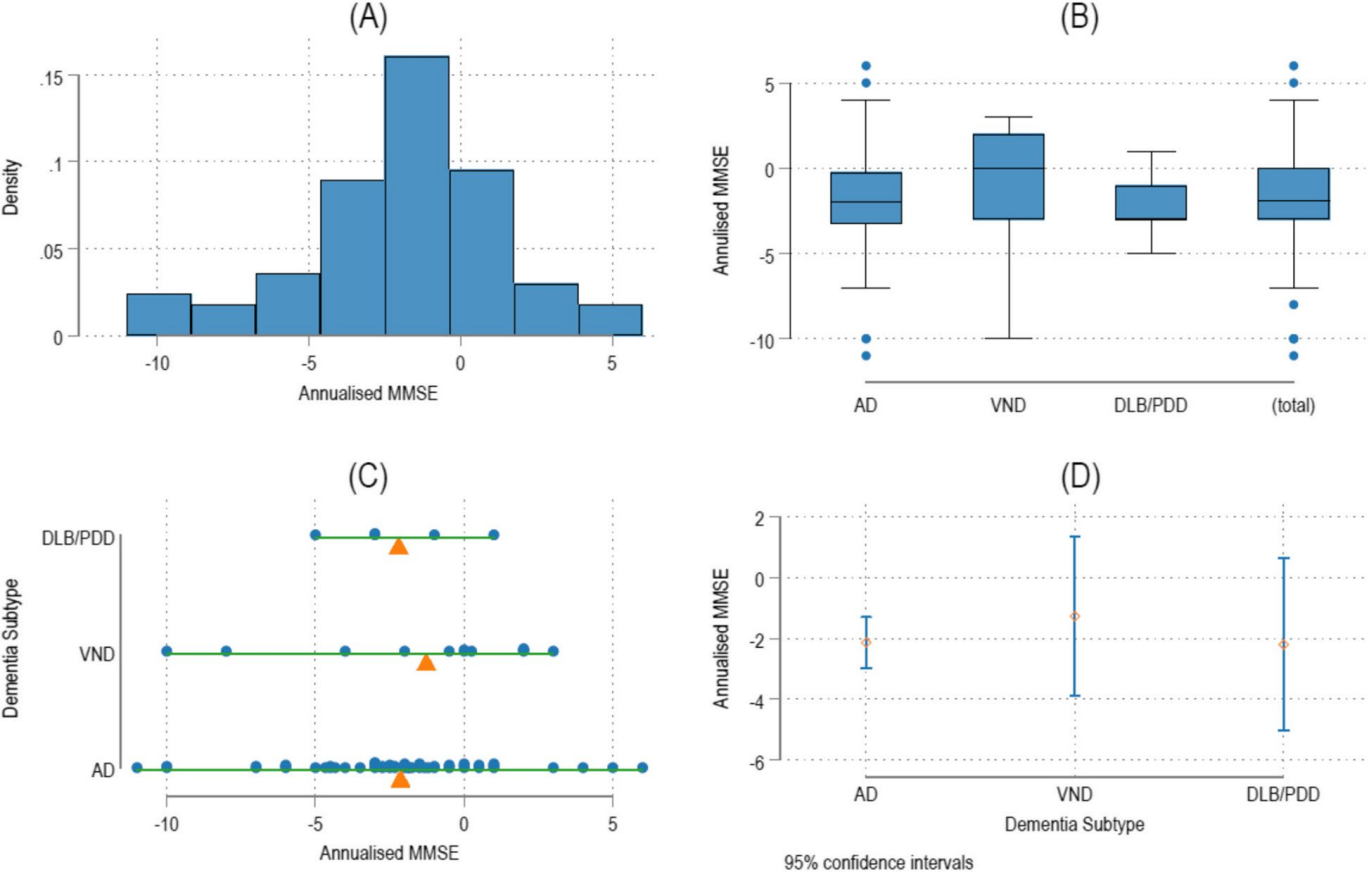
Annualised cognitive decline among the dementia subtypes. Abbreviations: MMSE, Mini-Mental State Examination; AD, Major Neurocognitive Disorder due to Alzheimer’s disease; VND, Major Vascular Neurocognitive Disorder; DLB, Dementia with Lewy bodies; PDD, Parkinson’s disease dementia; FTD, Fronto-temporal dementia

Figure 2 shows assessments for annualised cognitive decline among the dementia subtypes. The histogram (A) shows that there were no serious deviations from normality. We were therefore able to use the mean MMSE to reflect the drop in annual MMSE points. The beam (C) and confidence interval (D) plots show annual drop in MMSE points of 2.2, 2.1 and 1.3 of participants with DLB/PDD, AD, and VND respectively. These results should be interpreted with caution since 26 participants had one baseline MMSE score and were not analyzed for cognitive decline.

Table 5 shows mixed effects modelling for MMSE cognitive scores. Cognitive scores differed significantly for those with 13 years and above of education. They had higher MMSE scores compared to participants with less than 8 years of education in the three different mixed effects modeling which included either TTE (4.07; 95% C.I. 1.90, 6.25; p < 0.0001), age at symptom onset (4.15; 95% C.I. 2.00, 6.31; p < 0.0001) or age at dementia diagnosis (4.27; 95% C.I. 2.11, 6.43; p < 0.0001). Cognitive scores also differed significantly for VND compared with AD. The VND group had higher MMSEs on all three mixed effects models: TTE (2.37; 95% C.I. 0.43, 4.31; *p* = 0.017), age at symptom onset (2.81; 95% C.I. 0.88, 4.74; *p* = 0.004) and age at dementia diagnosis (2.82; 95% C.I. 0.87, 4.77; *p* = 0.005).

**Table 5:** Mixed effects modelling of factors associated with mini-mental state examination scores

Model 1 with TTE				Model 2 with Age at Onset				Model 3 with Age at Diagnosis			
	**Coeff**	**95% CI**	***p*-value**		**Coeff**	**95% CI**	***p*-value**		**Coeff**	**95% CI**	***p*-value**
**TTE**	-0.37	-1.19—0.45	0.375	**Age at Onset**	0.80	-0.62—2.22	0.270	**Age at Diagnosis**	0.91	-0.67—2.49	0.260
**TTE**^**2**^	0.02	-0.03—0.06	0.482	**Age at Onset**^**2**^	-0.005	-0.01—0.05	0.339	**Age at diagnosis**^**2**^	-0.01	-0.02—0.05	0.299
**Sex**				**Sex**				**Sex**			
***Male***	-0.48	-1.87—0.91	0.500	***Male***	-0.15	-1.52—1.22	0.826	***Male***	-0.19	-1.55—1.81	0.789
**Years of Education**				**Years of Education**				**Years of Education**			
**8 to 12 years**	1.38	-0.14—2.90	0.075	**8 to 12 years**	1.34	-0.15—2.84	0.078	**8 to 12 years**	1.45	-0.04—2.95	0.057
** ≥ 13 years**	4.07	1.90—6.25	** < 0.0001**	** ≥ 13 years**	4.15	2.00—6.31	** < 0.0001**	** ≥ 13 years**	4.27	2.11—6.43	** < 0.0001**
**Comorbidity Score**				**Comorbidity Score**				**Comorbidity Score**			
**3 to 4**	0.95	-0.51—2.41	0.201	**3 to 4**	0.83	-0.62—2.28	0.259	**3 to 4**	0.95	-0.50—2.39	0.200
** ≥ 5**	1.63	-0.04—3.31	0.056	** ≥ 5**	1.44	-0.23—3.10	0.091	** ≥ 5**	1.50	-0.13—3.14	0.072
**Dementia Subtype**				**Dementia Subtype**				**Dementia Subtype**			
**VND**	2.37	0.43—4.31	**0.017**	**VND**	2.81	0.88—4.74	**0.004**	**VND**	2.82	0.87—4.77	**0.005**
**DLB/PDD**	-0.16	-2.64—2.32	0.901	**DLB/PDD**	0.05	-2.34—2.44	0.968	**DLB/PDD**	0.07	-2.34—2.49	0.951
**FTD**	0.25	-4.80—5.30	0.923	**FTD**	0.93	-4.08—5.94	0.716	**FTD**	0.39	-4.63—5.42	0.878

*Abbreviations*: *TTE* Time to Event = from onset of symptoms to death or end of study period, *Coeff* Coefficient, *CI* confidence interval, *Age at Onset* Age at onset of symptoms of dementia, *Age at Diagnosis* Age when diagnosis of dementia was established, *TTE*^*2*^ TTE-squared variables, *Age at Onset*^*2*^ age at onset-squared variables, *Age at diagnosis*^*2*^ age at diagnosis-squared variables, *VND* Major Vascular Neurocognitive Disorder, *DLB* Dementia with Lewy bodies, *PDD* Parkinson’s disease dementia, *FTD* Fronto-temporal dementia

### Survival

Table 6 shows survival characteristics of participants according to dementia subtype. Of the 165 participants, 112 (67.9%) died during the study period. The mean age at death of all participants with dementia was 77.3 years (SD = 7.3) and was highest for AD (78.9 years, SD = 7.4). Using the Kruskal–Wallis test, there was a significant difference between the dementia subtype groups for mean age at death (χ^2^(4) = 13.51, *p* = 0.009). The Mann–Whitney test showed that the mean age at death was only significantly different between AD and VND (*p* = 0.001). The mean survival time of all deceased participants with dementia was 6.7 years (SD = 3.4), being highest for AD (7.3 years, SD = 3.5) and least with mixed dementia (4.1 years, SD = 0.2) followed by DLB/PDD (4.8 years, SD = 2.6). There was a statistically significant difference among the dementia subtype groups (χ^2^(4) = 11.15, *p* = 0.025). The Mann–Whitney test showed a significant difference of the mean survival time only between AD and DLB/PDD (*p* = 0.004).

**Table 6:** Survival characteristics of participants according to dementia subtype

Characteristics	All (*n* = 165)	AD (*n* = 117)	VND (*n* = 24)	DLB/PDD (*n* = 11)	FTD (*n* = 3)	Mixed (*n* = 4)	F-test	p-value
**Number of deaths**	112	79	16	10	3	2	4.69^a^	0.321
(%)	67.9	67.5	66.7	90.9	100	50	0.17^a^	0.334
**Mean Age at Death**	**(*n* = 112)**	**(*n* = 79)**	**(*n* = 16)**	**(*n* = 10)**	**(*n* = 3)**	**(*n* = 2)**		
(SD)	77.3 (7.3)	78.9 (7.4)	73.5 (5.7)	76.1 (5.6)	72 (7.2)	73 (0)	13.51^b^	0.009
**Mean Follow-up time** Years (SD)	7.2 (3.3)	7.8 (3.4)	5.8 (2.6)	4.96 (2.5)	7.7 (1.9)	6.3 (4.4)	15.39^b^	0.004
**Mean survival time (Deceased)**	**(*n* = 111)**	**(*n* = 78)**	**(*n* = 16)**	**(*n* = 10)**	**(*n* = 3)**	**(*n* = 2)**		
Years (SD)	6.7 (3.4)	7.3 (3.5)	5.5 (2.9)	4.8 (2.6)	7.7 (1.9)	4.1 (0.2)	11.15^b^	0.025

^a^chi-square value for the chi-square test of independence to compare two categorical variables

^b^Kruskal-Wallis tests: non-normally distributed data

*Abbreviations*: *n* number, *SD* Standard Deviation, *AD* Major Neurocognitive Disorder due to Alzheimer’s disease, *VND* Major Vascular Neurocognitive Disorder, *DLB* Dementia with Lewy bodies, *PDD* Parkinson’s disease dementia, *FTD* Fronto-temporal dementia

**Fig. 3:**
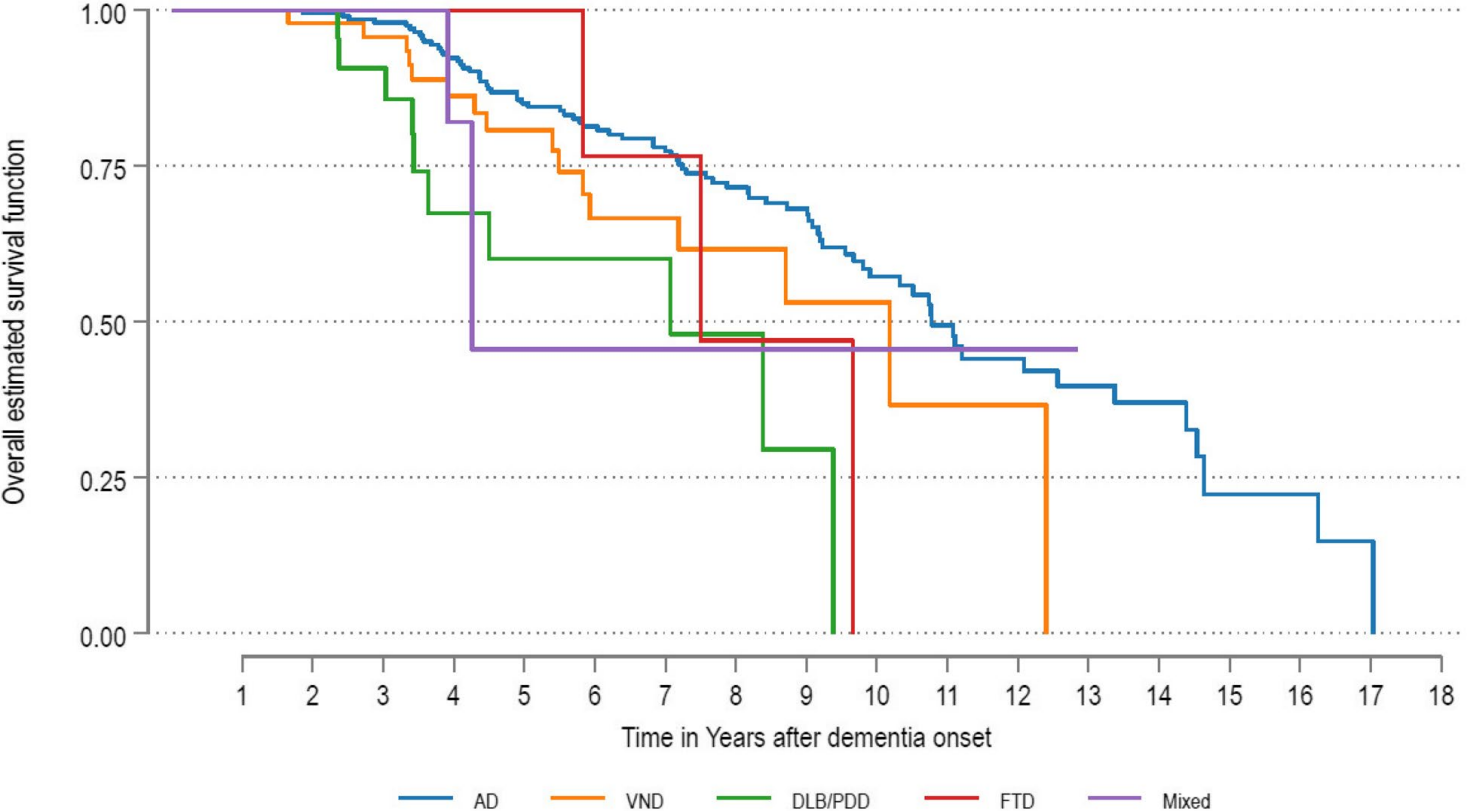
Age and sex adjusted survival rates of dementia subtypes. Abbreviations: AD, Major Neurocognitive Disorder due to Alzheimer’s disease; VND, Major Vascular Neurocognitive Disorder; DLB, Dementia with Lewy bodies; PDD, Parkinson’s disease dementia; FTD, Fronto-temporal dementia, Mixed = Mixed dementia

Figure 3 shows the survival curves for the different groups of dementia subtypes. A log rank test showed significantly different survival among the different dementia subtypes (χ^2^(4) = 18.03) with a p-value = 0.0001. Survival was only significantly different between AD and DLB/PDD participants, log rank test (χ^2^ (1) = 15.31) and p-value = 0.0001 with AD having a longer survival.

Table 7 shows the results of univariate survival analysis. Women had a longer survival compared to the men (HR = 0.59, 95% C.I. 0.40, 0.86, *p* = 0.006). Participants with Charlson’s Weighted Comorbidity scores of 5 and above had a shorter survival than those with scores 1 to 2 (HR = 1.81, 95% C.I. 1.13, 2.89, *p* = 0.013). VND group had a shorter survival than AD (HR = 1.83, 95% C.I. 1.06, 3.18, *p* = 0.03). Participants with DLB/PDD also had a significantly shorter survival compared to AD (HR = 3.55, 95% C.I. 1.82, 6.97, *p* < 0.001).

**Table 7:** Univariate analysis of baseline characteristics and survival

Characteristics	Alive	Dead	HR (95% CI)	*p* value
**Age at onset (years)**				
< 65 years	17 (32.1)	29 (26.1)	1	
> 65 years	36 (67.9)	82 (73.9)	1.41 (0.92—2.16)	0.117
**Gender: n (%)**				
Male	18 (34.0)	46 (41.1)	1	
Female	35 (66.0)	66 (58.9)	0.59 (0.40—0.86)	**0.006**
**Marital Status: n (%)**				
Divorced	4 (7.6)	16 (14.3)	1	
Married	35 (66.0)	56 (50.0)	0.86 (0.49—1.50)	0.596
Single	0	7 (6.3)	2.05 (0.84—5.02)	0.115
Widowed	14 (26.4)	33 (29.5)	0.72 (0.39—1.32)	0.291
**Years of Education: n (%)**				
0 to 7	13 (25.0)	26 (23.2)	1	
8 to 12	31 (59.6)	74 (66.1)	1.37 (0.86—2.17)	0.181
> 12	8 (15.4)	12 (10.7)	1.16 (0.58—2.34)	0.671
**Charlson's Weighted Comorbidity score**				
1 to 2	27 (50.9)	39 (34.8)	1	
3 to 4	16 (30.2)	39 (34.8)	1.43 (0.91—2.24)	0.117
≥ 5	10 (18.9)	34 (30.4)	1.81 (1.13—2.89)	**0.013**
**Baseline MMSE**				
Median (IQR)	22 (5)	21 (6)	0.97 (0.94—1.01)	0.201
**MAP**				
Median (IQR)	99.3 (18.3)	101.7 (16.7)	1.01 (0.99—1.03)	0.512
Mean (SD)	101.0 (14.0)	103.5 (14.5)		
**Dementia subtype**				
AD	38 (77.6)	79 (71.8)	1	
VND	8 (16.3)	16 (14.6)	1.83 (1.06—3.18)	**0.030**
DLB/PDD	1 (2.0)	10 (9.1)	3.55 (1.82—6.97)	** < 0.001**
FTD	0	2 (2.7)	1.77 (0.56—6.65)	0.334
Mixed	2 (4.1)	2 (1.8)	1.01 (0.25—4.15)	0.986

*Abbreviations*: *n* number, *SD* Standard Deviation, *IQR* Inter Quartile Range, *MMSE* Mini-Mental State Examination, *MAP* Mean Arterial Pressure, *HR* Hazard Ratio, *CI* confidence interval, *AD* Major Neurocognitive Disorder due to Alzheimer’s disease, *VND* Major Vascular Neurocognitive Disorder, *DLB* Dementia with Lewy bodies, *PDD* Parkinson’s disease dementia, *FTD* Fronto-temporal dementia

Table 8 shows the results of the multivariate Cox regression survival analysis. Age at symptom onset greater than 65 compared to age at symptom onset 65 or younger was associated with shorter survival (HR = 1.82, 95% C.I. 1.11, 2.99, *p* = 0.017). Female gender was associated with increased survival compared to males (HR = 0.59, 95% C.I. 0.36, 0.95, *p* = 0.029). Comorbidity scores of 3 to 4 (HR = 1.88, 95% C.I. 1.14, 3.10, *p* = 0.014) and scores of 5 or more (HR = 1.97, 95% C.I. 1.16, 3.34, *p* = 0.012) were associated with shorter survival compared to scores of 1 to 2. Lower baseline MMSE was associated with shorter survival (HR = 1.05, 95% C.I. 1.01, 1.10, *p* = 0.029). Survival decreased by 5% for every one unit decrease in MMSE. The group with DLB/PDD had shorter survival compared to those with AD (HR = 3.07, 95% C.I. 1.50, 6.29, *p* = 0.002). Harrell's C concordance statistic was 0.681.

**Table 8:** Predictors of survival among patients with dementia—Multivariate cox regression analysis

	**Std err**	**z**	**HR**	**95% CI**	***p*-value**
**Age at onset**					
> 65 years	0.46	2.38	1.82	1.11—2.99	**0.017**
**Gender**					
Female	0.14	-2.18	0.59	0.36—0.95	**0.029**
**Marital Status**					
Married	0.26	-0.44	0.86	0.48—1.58	0.660
Single	0.99	1.44	2.02	0.78—5.26	0.149
Widowed	0.23	-1.16	0.67	0.34—1.32	0.246
**Years of Education**					
8 to 12	0.40	1.65	1.54	0.92—2.57	0.098
≥ 13	0.42	0.24	1.10	0.51—2.33	0.813
**Charlson's Weighted Comorbidity score**					
3 to 4	0.48	2.46	1.88	1.14—3.10	**0.014**
≥ 5	0.53	2.52	1.97	1.16—3.34	**0.012**
**Baseline MMSE**					
Median (IQR)	0.02	2.18	1.05	1.01—1.10	**0.029**
**Dementia subtype**					
VND	0.44	0.91	1.34	0.71—2.55	0.365
DLB/PDD	1.12	3.07	3.07	1.50—6.29	**0.002**
FTD	1.06	0.74	1.63	0.45—5.87	0.458
Mixed	1.28	0.74	1.73	0.41—7.36	0.457

*Abbreviations*: *n* number, *IQR* Inter Quartile Range, *MMSE* Mini-Mental State Examination, *HR* Hazard Ratio, *CI* confidence interval, *VND* Major Vascular Neurocognitive Disorder, *DLB* Dementia with Lewy bodies, *PDD* Parkinson’s disease dementia, *FTD* Fronto-temporal dementia

## Discussion

In our study, the commonest dementia subtype was AD followed by VND. The overall duration from symptom onset until diagnosis (date of first clinic visit) was 2.5 years. The combined DLB/PDD subtype group had the highest age of symptom onset while AD had the highest age at diagnosis. Cognitive scores were significantly higher for VND compared to AD subtypes and higher for participants with longer duration of education. Cognitive decline was faster in the DLB/PDD subtype and in the AD group compared with VND. Survival in the DLB/PDD group was lower compared to the AD group. Other factors significantly associated with reduced survival were older age of dementia onset, lower baseline cognition, and higher comorbidity scores. Female gender was associated with increased survival.

We found AD to be the commonest dementia subtype (70.9%), followed by VND (14.6%). Our results are not consistent with other studies as our AD frequency was higher [4, 28, 29]. In memory clinic or hospital-based studies from different parts of the world, the prevalence of AD ranged between 38 and 67% and 5% to 26% for VND [4, 28–31]. Our findings of a higher proportion of AD could relate to how we categorized AD subtype. For example, participants with combinations of probable AD plus possible VND or participants with probable AD plus possible DLB were all classified primarily as AD. Differences in proportions of our dementia subtypes could be influenced by variations in criteria to categorize dementia and differences in the interpretation of these criteria. Findings of the Lewy Body-containing dementias of DLB (3.6%) and PDD (3%) as the third commonest dementia subtype have been shown in previous memory clinic studies [28, 31]. Patients presenting with motor symptoms of Parkinsonism in this hospital are usually channeled to the neurology clinic resulting in fewer Lewy Body-containing dementias at our memory clinic. Our finding of a prevalence of FTD of 1.8% is similar to a cohort from a memory clinic in Hong Kong of a comparable mean age at diagnosis as our study of 76.1 years [28]. Findings from a large memory clinic cohort in France with a mean age of 56 years found a higher prevalence of FTD of 9.7% [32]. The low frequency of FTD in our study could therefore be due to the higher inclusion criterion age cut-off of 60 years. Patients with initial behavioral symptoms with FTD could have been referred to the psychiatric clinic and not the memory clinic.

The highest age of symptom onset of 70.9 years in our study was in the DLB/PDD group. Amoo et al. in study of a Nigerian hospital cohort found the AD group to have the highest mean age of symptom onset of 72.8 years compared to DLB patients with a mean age of symptom onset of 65 years [4]. It is unclear from the publication whether the cohort comprised outpatients, in-patients, or both. Our age of symptom onset for DLB/PDD dementias is, however, similar to the findings of a recent Chinese study which reported a mean age of symptom onset of 68.6 years [33]. As stated above, our DLB/PDD group is biased group as many younger DLB/PDD patients are seen in the neurology clinic.

Our study found an overall duration of illness of 30.6 months, from symptom onset to diagnosis. This is comparable to memory clinic study findings in India and Hong Kong [28, 29]. A lower duration of 13.8 months was found in an Italian study [34]. In the developed world, organizational challenges of memory clinics coupled with long waiting lists are thought to explain the longer duration from symptom onset to diagnosis [35]. Our challenges of long waiting lists due to fewer qualified personnel in the memory clinic are similar to findings elsewhere [36]. Also in our context, significant functional impairment is often the trigger for caregivers to seek medical help, and this usually occurs late [34, 37]. In our cohort, duration from symptom onset to diagnosis could have been shortest in VND (22.9 months) followed by those with DLB/PDD (26.2 months) because of the earlier motor or other non-cognitive symptoms that lead to patients or caregivers recognising the illness sooner. In our setting, early symptoms of AD are more likely to be seen as “old age” until patients become very functionally impaired and present late to the clinic.

The dementia subtype with the highest age at diagnosis of 73.6 years was AD. In addition to early cognitive symptoms being attributed to “old age” leading to diagnosis later in the disease trajectory, our inclusion criterion of age 60 years and above and the triage of some people to the geriatric clinic could have contributed to an exclusion bias of people with young onset dementia and older frail people with advanced dementia [38].

The mean annual rate of cognitive decline of MMSE points per dementia subtype in our study was 2.2 for the group with DLB/PDD, 2.1 for AD and 1.3 for VND. The decline in cognition between DLB and PDD is similar to that shown by a Swedish study [39]. We considered DLB and PDD as one group in the analyses due to the small numbers in each of these groups. DLB and PDD may, in any case, be considered on the same spectrum of pathological disorders [40]. In our study, cognitive decline of DLB/PDD and AD were similar, a finding different to a multi-centre cohort study [6]. The similar annual rates of decline between DLB/PDD and AD in our study could be due to the combination of AD + DLB pathologies which we could have classified as AD. Studies have shown that dual AD + DLB pathology has a faster cognitive decline than either individual dementia subtypes [14, 41–43].

We found significantly higher MMSE scores for VND compared to AD, findings similar to those of a Canadian study [44]. In our study, we used the MMSE, an inferior tool for detecting subcortical dysexecutive cognitive related impairment, hence the higher VND scores [45]. The Montreal Cognitive Assessment (MoCA) was not universally used until the later years of the study period.

Thirteen or more years compared to less than eight years of education was also associated with significantly higher MMSE scores in our study. Education levels have been shown to affect performance of cognitive tests like MMSE [46]. Higher levels of education have been associated with higher MMSE scores in both developed and developing countries [46–48]. Majority of our participants could have had high MMSE scores due to the higher literacy rate of participants as indicated by the MMSE score criteria in the triage of who would be seen in the memory or geriatric clinics. The high education levels of 76% (Table 1) of participants having eight or more years of education may be as a result of memory clinic population catchment area in the Western Cape province of South Africa having the highest literacy rates in South Africa of approximately 80% [49].

The finding of the DLB/PDD group having a higher mortality compared to AD is consistent with previous studies [50, 51]. We did not explore possible causes of death, but a previous study has shown that fall-related injuries and pneumonias contributed to mortality [50]. Shorter survival due to onset of dementia symptoms ≥ 65 years has also been shown in studies elsewhere [17, 52]. Increased mortality due to late onset of dementia symptoms could be due to increased vulnerability to infections due to aspiration pneumonia and urinary tract infections, injuries related to poor mobility, and adverse reactions due to psychotropic and sedative medicines, the choice of which is limited particularly in the public healthcare service. The infections and injuries are associated with progression of the dementia syndrome as a result of excess damage accumulation or rapidly shrinking resilience due to accelerated aging of the participants [53, 54]. We found a large proportion (67.9%) of our cohort died during the follow-up period with a mean survival time of 6.7 years. This finding is higher than mortality of 57.4% of 779 dementia patients in an Australian study carried out in nine memory clinics [17]. This difference could be due to late diagnosis in our cohort compared to the earlier dementia diagnosis in the Australian cohort which is associated with longer survival similar to findings in the Dutch study which focused on early onset dementia [19]. Majority (61.6%) of the Australian PRIME study cohort were on cholinesterase inhibitors which could have slowed cognitive decline and probably reduced mortality rate compared to our cohort [55]. Females were associated with increased survival compared to males, a finding again consistent with other studies [15, 17]. In our study, survival decreased by 5% for every one unit decrease in MMSE score. This is consistent with the findings from similar published studies showing shorter survival with a lower MMSE scores [15, 56, 57]. The short survival in our study population could have been due to increased risk of infections. We did not have data on the causes of death as registered on the death certificate, which are commonly reported as natural cause particularly in frail patients with dementia. The risk of infection was increased in our study population because the care is provided by family and other caregivers who have little knowledge and no formal support in the care of patients with swallowing disorders and/or bladder and bowel dysfunction and poor mobility. With our study showing reduced survival with reduced cognitive score, comorbidity burden has been associated with impaired cognitive performance and decline [58, 59]. Higher comorbidity as characterized by Charlson’s comorbidity index scores of 3 to 4 and 5 and above in our study, was associated with higher mortality among dementia patients. Previous studies have assessed comorbidity differently [57, 60, 61]. Our study relied on the documented comorbidities limited to Charlson’s index tool [26]. We therefore did not consider geriatric syndromes and other conditions not in the Charlson’s comorbidity index that could also influence survival.

To our knowledge, this is the first published longitudinal study carried out in a memory clinic in Africa, describing dementia subtypes, cognitive decline, and survival over a 10-year period. The strengths of the study include the categorization of dementia using validated diagnostic criteria as well as obtaining complete survival data with a mean follow up period of 7.2 years. The study cohort was, however, a specific group of people referred to a memory clinic and so the results are not generalizable to all people with dementia in the community.

The study was retrospective. We depended on the data collected at the time and the clinicians’ notes. Clearly a prospective study with a data collection protocol set up in advance would have considerably reduced the number of exclusions (93 in total) we had to make for missing data. We excluded 21 patients with advanced dementia (Fig. 3). These exclusions would have affected dementia sub-type proportions and survival outcomes. However, determining dementia sub-type in advanced disease would be difficult anyway. The use of MMSE has several limitations including floor and ceiling effects as well as cultural and linguistic validity concerns.

Another important limitation was not having clinical diagnoses validated by autopsy which is the ultimate reference standard for dementia diagnosis. However, we used diagnostic criteria that have been validated in some post-mortem brain studies [62].

## Conclusion

In conclusion, we have reported comparable proportions of dementia subtypes and their characteristics from this 10-year longitudinal memory clinic cohort in South Africa. We describe cognitive decline of some dementia subtypes and factors affecting cognitive scores such as dementia subtype and education level. There was a high death rate in this cohort, comparable to other similar populations. The factors associated with shorter survival included DLB/PDD group, older age of symptom onset, lower baseline cognition, and higher comorbidity scores. Females were associated with increased survival.

Future longitudinal studies in Africa could explore dementia subtype proportions for younger onset dementia subtypes and cognitive decline of specific dementia subtypes like FTD, which we were unable to analyze due to a smaller number of participants. There is a further need to assess other known predictors of mortality like neuropsychiatric symptoms, polypharmacy, and functional impairment including how activity of daily living scores or carer burden change over time in larger longitudinal memory clinic studies in Africa. An early reliable dementia subtype diagnosis and knowledge of survival outcomes is important where complex investigations may be lacking but where potential disease-modifying therapies may become available in the future.

### Supplementary Information

**Additional file 1:** **Figure S1. **Distribution of data using box plots for baseline MMSE, baseline mean arterial pressures, TSH and vitamin B12 serum levels, by dementia subtype. **Figure S2.** Patterns of decline of mean MMSE scores at clinic visits by dementia subtype. **Figure S3.** Change in mean MMSE scores of participants with AD and VND who had more than one annual score recorded.

## Data Availability

All data generated or analyzed during this study are included in this published article (and its supplementary information files).

## Abbreviations

AD: Major Neurocognitive Disorder due to Alzheimer’s disease
DLB: Dementia with Lewy Body
DSM: Diagnostic and Statistical Manual of Mental Disorders
FTD: Fronto-temporal dementia
HIV: Human Immuno-deficiency Virus
MMSE: Mini-Mental State Examination
MoCA: Montreal Cognitive Assessment
PDD: Parkinson disease dementia
RBANS: Repeatable Battery for the Assessment of Neuropsychological Status
TTE: Time to Event
VND: Major Vascular Neurocognitive Disorder
WHO: World Health Organisation
