# Supplementary material for: Dementia subtypes, cognitive decline and survival among older adults attending a memory clinic in Cape Town, South Africa: a retrospective study
Source: BMC Geriatr. 2023 Dec 9;23:829. doi: 10.1186/s12877-023-04536-3 (PMC10709983; doi:10.1186/s12877-023-04536-3)
Supplement: Supplementary file 1 — Additional file 1: Figure S1. Distribution of data using box plots for baseline MMSE, baseline mean arterial pressures, TSH and vitamin B12 serum levels, by dementia subtype. Figure S2. Patterns of decline of mean MMSE scores at clinic visits by dementia subtype. Figure S3. Change in mean MMSE scores of participants with AD and VND who had more than one annual score recorded. [file 12877_2023_4536_MOESM1_ESM.docx]

**Supplementary Figure 1; Distribution of data using box plots for baseline MMSE, baseline mean arterial pressures, TSH and vitamin B12 serum levels, by dementia subtype.**

*Abbreviations:MMSE, Mini-Mental State Examination; MAP, Mean Arterial Pressure; TSH, Thyroid Stimulating Hormone – Normal range = 0.38 to 5.33 mIU/L; Vitamin B12 – Normal range =* *145 to 569 pmol/L; AD, Major Neurocognitive Disorder due to Alzheimer’s disease; VND, Major Vascular Neurocognitive Disorder; DLB, Dementia with Lewy bodies; PDD, Parkinson’s disease dementia; FTD, Frontotemporal Dementia; Mixed; Mixed dementia*

**Supplementary Figure 2; Patterns of decline of mean MMSE scores at clinic visits by dementia subtype.**

*Abbreviations: MMSE, Mini-Mental State Examination; AD, Major Neurocognitive Disorder due to Alzheimer’s disease; VND, Major Vascular Neurocognitive Disorder; DLB, Dementia with Lewy bodies; PDD, Parkinson’s disease dementia; FTD, Frontotemporal Dementia; Mixed, Mixed dementia.*

**Supplementary Figure 3; Change in mean MMSE scores of participants with AD and VND who had more than one annual score recorded.**

*Abbreviations: MMSE, Mini-Mental State Examination; AD, Major Neurocognitive Disorder due to Alzheimer’s disease; VND, Major Vascular Neurocognitive Disorder.*

*NB:* *The two largest groups (AD and VND), had a large proportion with not more than one MMSE score (i.e., baseline score only). Therefore, the results shown in figures 2 and 3 should be interpreted with caution.*
